# Endogenous progesterone levels and frontotemporal dementia: modulation of TDP-43 and Tau levels *in vitro* and treatment of the A315T *TARDBP* mouse model

**DOI:** 10.1242/dmm.011460

**Published:** 2013-06-20

**Authors:** Theresa N. T. Dang, Carol Dobson-Stone, Elias N. Glaros, Woojin S. Kim, Marianne Hallupp, Lauren Bartley, Olivier Piguet, John R. Hodges, Glenda M. Halliday, Kay L. Double, Peter R. Schofield, Peter J. Crouch, John B. J. Kwok

**Affiliations:** 1 Department of Pathology, The University of Melbourne, Victoria 3010, Australia; 2 Neuroscience Research Australia, Sydney, NSW 2031, Australia; 3 School of Medical Sciences, University of New South Wales, Sydney, NSW 2052, Australia; 4 Centre for Vascular Research, University of New South Wales, Sydney, NSW 2052, Australia; 5 Florey Institute of Neuroscience and Mental Health, The University of Melbourne, Melbourne, Victoria 3010, Australia

## Abstract

Frontotemporal dementia (FTD) is associated with motor neurone disease (FTD-MND), corticobasal syndrome (CBS) and progressive supranuclear palsy syndrome (PSPS). Together, this group of disorders constitutes a major cause of young-onset dementia. One of the three clinical variants of FTD is progressive nonfluent aphasia (PNFA), which is focused on in this study. The steroid hormone progesterone (PROG) is known to have an important role as a neurosteroid with potent neuroprotective and promyelination properties. In a case-control study of serum samples (39 FTD, 91 controls), low serum PROG was associated with FTD overall. In subgroup analysis, low PROG levels were significantly associated with FTD-MND and CBS, but not with PSPS or PNFA. PROG levels of >195 pg/ml were significantly correlated with lower disease severity (frontotemporal dementia rating scale) for individuals with CBS. In the human neuroblastoma SK-N-MC cell line, exogenous PROG (9300–93,000 pg/ml) had a significant effect on overall Tau and nuclear TDP-43 levels, reducing total Tau levels by ∼1.5-fold and increasing nuclear TDP-43 by 1.7- to 2.0-fold. Finally, elevation of plasma PROG to a mean concentration of 5870 pg/ml in an Ala315Thr (A315T) *TARDBP* transgenic mouse model significantly reduced the rate of loss of locomotor control in PROG-treated, compared with placebo, mice. The PROG treatment did not significantly increase survival of the mice, which might be due to the limitation of the transgenic mouse to accurately model TDP-43-mediated neurodegeneration. Together, our clinical, cellular and animal data provide strong evidence that PROG could be a valid therapy for specific related disorders of FTD.

## INTRODUCTION

Frontotemporal dementia (FTD) is the most common type of presenile dementia (onset under 65 years) and the fourth most common type of dementia (Ratnavalli et al., 2002). Clinically, FTD represents a heterogeneous group of clinical syndromes defined by early personality and behavioural changes or progressive aphasia, and includes the clinical variant progressive nonfluent aphasia (PNFA) (Rascovsky et al., 2011; Gorno-Tempini et al., 2011). Corticobasal syndrome (CBS) and progressive supranuclear palsy syndrome (PSPS) are closely related to FTD both in terms of clinical symptoms and underlying pathology, and are often included within the broader spectrum (Boeve, 2007). Motor neurone disease (MND), also known as amyotrophic lateral sclerosis (ALS), is characterised by degeneration of upper and lower motor neurons, leading to progressive weakness and muscle atrophy with eventual paralysis and death within 5 years of clinical onset (Boillée et al., 2006). MND also overlaps with FTD, with at least 15% of FTD patients developing MND and vice versa (FTD-MND) (Lillo and Hodges, 2009; Burrell et al., 2011).

The neuropathology of FTD can include the presence of degenerating neurons containing intracellular inclusions positive for either the microtubule-associated protein Tau (MAPT), TAR DNA-binding protein (TDP-43), a highly conserved heteronuclear ribonuclearprotein (hnRNP) or other ubiquitylated proteins (reviewed in Mackenzie et al., 2010). The *MAPT* gene was the first FTD locus to be identified (reviewed in Sieben et al., 2012), and mutations in the gene encoding TDP-43 (*TARDBP*) can give rise to MND (Sreedharan et al., 2008) and FTD in rare instances (Borroni et al., 2010). Clinicopathological correlations have revealed that certain clinical subgroups are associated predominantly with either Tau or TDP-43 neuropathology, with all individuals with FTD-MND having TDP-43 immunopositive inclusions and most individuals with CBS, PSPS and PNFA having Tau inclusions at autopsy (Hodges et al., 2004; Schofield et al., 2012).

The steroid hormone progesterone (PROG) is best known for its role in female reproduction, although it also has an important role as a neurosteroid with potent neuroprotective and promyelinating pathways (Stein, 2011). In neurodegenerative diseases, PROG has been shown to be a potential treatment for MND (Singh and Su, 2013). Elevated serum PROG levels have been associated with milder MND symptoms and later age at onset, with PROG suggested to protect against oxidative damage (Gargiulo Monachelli et al., 2011). Moreover, PROG has been demonstrated to successfully reverse impaired motor phenotypes in the Wobbler genetic model of MND (Gonzalez Deniselle et al., 2002), which carries a mutant *Vps54* gene (Schmitt-John et al., 2005). In this study, we examined the relationship between serum PROG levels and disease status and symptom severity in FTD patients with predictable pathology. We then examined whether exogenous PROG could modulate the levels of TDP-43 and Tau in an *in vitro* model using a human neuroblastoma cell line. Finally, we examined whether PROG implants could modify the phenotype of a mouse model carrying the Ala315Thr missense mutation of the *TARDBP* gene (TDP43^A315T^) (Wegorzewska et al., 2009).

**TRANSLATIONAL IMPACT**

**Clinical issue**

Frontotemporal dementia (FTD) is a common cause of young-onset dementia. The condition is difficult to diagnose because of the heterogeneity of symptoms, which can include cognitive, movement and language difficulties. Several clinical syndromes are associated with FTD, including motor neurone disease (MND), corticobasal syndrome (CBS) and progressive supranuclear palsy syndrome (PSPS). A clinical variant of FTD includes progressive non-fluent aphasia (PNFA). At present, there are no FDA-approved medications indicated for FTD treatment. The steroid hormone progesterone, which is thought to have neuroprotective functions in addition to its role in the female reproductive system, has been identified as a potential therapy for MND. In line with this, high levels of progesterone have been shown to be associated with positive patient outcome, and the hormone has also been used successfully to improve impaired motor phenotypes in a genetic mouse model of MND. The role of progesterone in FTD development and therapy has not been previously investigated.

**Results**

The authors examined whether reduced levels of endogenous progesterone are associated with the disease in FTD patients and whether exogenous progesterone can modulate the levels of the two major proteins associated with FTD pathology, namely Tau and TDP-43 (encoded by the *TARDBP* gene) in a cellular model. They identified low or undetectable levels of serum progesterone in association with FTD, particularly with MND and CBS, but not for the other disorders, PSPS and PNFA. Lower progesterone levels correlated with severe disease in CBS patients. The authors also demonstrated that high physiological concentrations of exogenous progesterone, such as those present during the menstrual cycle and pregnancy, reduced overall levels of Tau and increased nuclear TDP-43 levels. Finally, the authors report that elevation of exogenous progesterone in an FTD mouse model expressing a mutated version of *TARDBP* significantly reduced the rate of loss of motor control, but had no effect on mortality.

**Implications and future directions**

These data indicate that, as reported for MND, progesterone has an effect on disease parameters in FTD. The authors suggest that the effects of progesterone are mediated through changes in cellular Tau and TDP-43 protein levels. Collectively, the clinical, cellular and animal data presented in this work suggest that progesterone might have therapeutic possibilities for certain clinical subgroups of FTD. However, these findings require validation in studies that involve larger sample sizes and longitudinal measurements of Tau and progesterone levels. Moreover, new and improved mouse models that accurately recapitulate all aspects of FTD pathophysiology are needed.

## RESULTS

### Correlation between PROG levels and disease status in FTD clinical subgroups

In this study we focused on PROG in male FTD patients (*n*=39) because there is less natural variation in the hormone levels in males compared with females (Table 1). Moreover, to aid in the interpretation of our data, we further restricted our FTD subgroups to those whose clinical symptoms are highly correlated with specific neuropathological subgroups: FTD-MND (*n*=12, probable TDP-43 neuropathology), CBS (*n*=10, probable Tau pathology), PSPS (*n*=7, probable Tau pathology) and PNFA (*n*=10, probable Tau pathology). As a comparison, we examined a cohort of neurologically normal males (*n*=91) with a mean and range of ages comparable to our FTD cohort (Table 1). As expected, the median serum PROG levels of both the normal and FTD groups of elderly male individuals were low (median normal=289 pg/ml, range ≤195 to 358 pg/ml; median FTD=223 pg/ml, range ≤195 to 975 pg/ml). These values are comparable to those reported by Gargiulo Monachelli et al. (Gargiulo Monachelli et al., 2011) for neurologically normal males (mean=470 pg/ml, range ∼200 to 800 pg/ml) and male MND patients (mean=660 pg/ml, range ∼100 to 1380 pg/ml). A proportion of individuals had undetectable levels of the neurosteroid (assay threshold at ≤195 pg/ml). PROG levels were recoded as a categorical factor where individuals were classed either as low-expressors (≤195 pg/ml) or expressors (>195 pg/ml). Age was a significant predictor of serum PROG levels for controls (standardised β=−0.22, *P*=0.012) where a younger age was associated with an increase in those expressing PROG, and so age was included as a covariate in subsequent analyses. Using logistic regression to examine the association between the ability of a patient to produce PROG (low-expressor compared with expressor) and disease status, we observed that PROG low-expressors were more likely to have disease in our FTD cohort overall [Exp(B)=2.6, adjusted *P*=0.037; Table 1]. Clinical subgroup analysis, to determine whether there were disease-specific effects, showed that PROG low-expressors were significantly associated with FTD-MND [Exp(B)=5.0, adjusted *P*=0.037] and CBS status [Exp(B)=5.9, adjusted *P*=0.037], but not with the other Tau-based subgroups, PSPS and PNFA (Table 1).

**Table 1.**
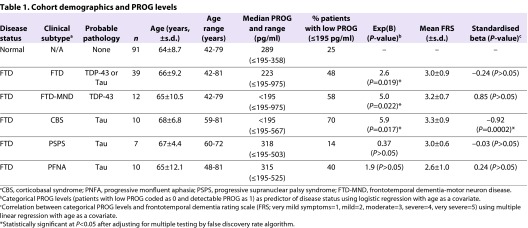
Cohort demographics and PROG levels

We further examined whether there was a relationship between PROG levels and disease severity in a subset of individuals with FTD (*n*=33) as determined by the frontotemporal dementia rating scale (FRS). Age was significantly correlated with FRS (standardised β=−0.41, *P*=0.016; data not shown) and was included as a covariate in subsequent analyses. Multiple linear regression analysis was used to examine the correlation between serum PROG levels and disease severity (Table 1). We observed that PROG levels were not significantly correlated with FRS when assessing all FTD patients (Table 1). However, in the subgroup analysis, PROG expressors (>195 pg/ml PROG) were significantly associated with lower FRS in CBS (*n*=6, standardised β=−0.92, adjusted *P*=0.001), but not for the FTD-MND, PNFA or PSPS subgroups (Table 1).

### Effects of PROG in cell culture and *in vivo*

**Fig. 1.**
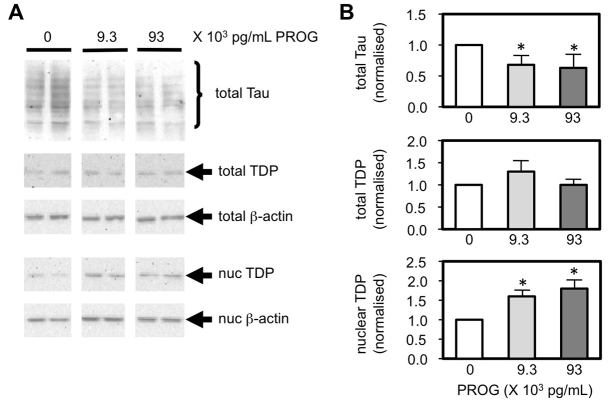
**Modulation of Tau and TDP-43 protein levels by PROG in SK-N-MC cells.** (A) Western blot analysis shows endogenous Tau and TDP-43 levels in the total and nuclear (nuc) fractions of cells treated with the neurosteroid. The housekeeping β-actin protein was used to normalise protein levels. (B) Chemiluminescent band intensities were quantified and the levels of total and nuclear proteins are presented relative to the untreated control cells. Mean and s.e.m. from *n*=4 independent experiments. Significance of *P*<0.05 is indicated (*).

We examined whether exogenous PROG could modulate the levels of two major pathological molecules, Tau and TDP-43, in a human neuroblastoma SK-N-MC cell culture model (Fig. 1). We observed that PROG had a significant effect on overall Tau levels, whereby both 9300 and 93,000 pg/ml of exogenous PROG significantly decreased (1.5-fold, *P*=0.034 for both concentrations) the level of total Tau protein compared with untreated cells. TDP-43 is a nuclear protein that normally translocates between the cytoplasm and nucleus. PROG did not significantly alter the total level of TDP-43, but both 9300 and 93,000 pg/ml of the neurosteroid significantly increased the level of TDP-43 in the nucleus (1.7- to 2.0-fold, *P*=0.034) compared with untreated cells (Fig. 1).

We examined the relationship between PROG and Tau levels in a subset of patients and controls for which serum Tau levels were available (*n*=3 controls, *n*=4 FTD). Multiple linear regression analysis showed that PROG level was a significant predictor of Tau level when an interaction term (disease status × PROG levels) was included in the regression equation to adjust for disease-specific changes in Tau levels (Sparks et al., 2012) (age standardised β=0.27, *P*=0.071; disease status standardised β=−1.44, *P*=0.004; PROG levels standardised β=−1.26, *P*=0.004; disease status × PROG levels standardised β=1.76, *P*=0.005; supplementary material Fig. S1).

### PROG treatment of the A315T TARDBP mutant mouse

**Fig. 2.**
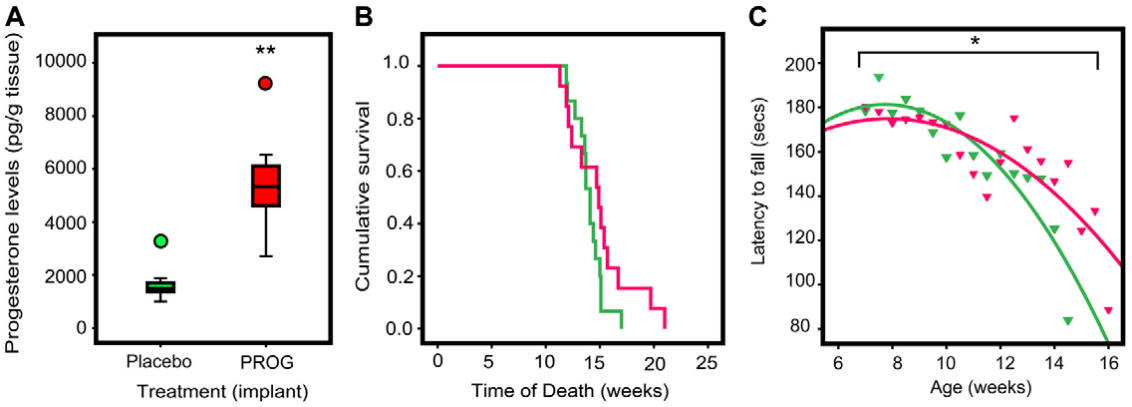
**Effect of PROG treatment on survival time and locomotor control of TDP43^A315T^ mutant mice.** (A) Levels of PROG in brain tissue of treated and untreated wild-type mice. (B) Survival time from day 0, with PROG pellet implantation at approximately week 6, to week 25 (at 100% mortality of entire cohort). Untreated TDP43^A315T^ mutant mice (green line, *n*=14 animals per group) were all deceased at week 17 compared with week 21 for treated mutant mice (pink line, *n*=13 animals per group; *P*>0.05). (C) Overall rate of change in locomotor control with increasing age was modelled as quadratic regression curves. The slope of the curve, which measures the decrease in time spent on the rotarod as the mice aged, was significantly decreased by PROG treatment of TDP43^A315T^ mutant mice (pink triangles) compared with untreated mutant (green triangles) mice. Significances of *P*<0.05 (*) and *P*<0.001 (**) are indicated.

We examined one of the first transgenic mouse lines to model mutant TDP-43-mediated neurodegeneration (Wegorzewska et al., 2009), in which the mutant Ala315Thr *TARDBP* gene is driven by the mouse prion promoter (Prp-TDP43^A315T^). In our experience, the male TDP43^A315T^ mouse had an average lifespan of 14.5 weeks (3.5 months) with maximum lifespan of 17.5 weeks (Fig. 2). Moreover, unlike other reported MND mouse models (reviewed in Tsao et al., 2012), a locomotor deficit was evident at a very early age when compared with non-transgenic littermates (6 weeks of age when phenotyping began), but this did not progress to end-stage immobility prior to the mice dying prematurely (our unpublished data). Moreover, levels of spinal cord choline acetyltransferase cholinesterase (ChAT), a marker for motor neurons, were not significantly different between wild-type and TDP43^A315T^ mice at the early stage of disease (supplementary material Fig. S2).

We examined whether treatment with PROG would alter locomotor deterioration or survival in the TDP43^A315T^ mouse by implanting PROG pellets designed to release PROG at an approximate rate of 2 mg PROG/day. We were able to confirm that PROG implants significantly elevated brain (mean PROG=5100 pg/g fresh brain tissue) and plasma (mean PROG=5870 pg/ml) levels in treated non-transgenic mice by 3.3- to 4.1-fold, respectively, compared with placebo-treated non-transgenic mice (*P*=0.001) at the end of the treatment period (Fig. 2A; supplementary material Fig. S3A). PROG-treated TDP43^A315T^ mice had a median survival of 14.9 weeks (with a maximal lifespan of 21.0 weeks) compared with placebo-treated TDP43^A315T^ mice, which had a median survival of 14.1 weeks (maximal lifespan of 17.0 weeks) (Fig. 2B). This apparent 5.6% increase in median survival (and 25% increase in maximal lifespan), however, did not reach statistical significance [*P*=0.155, log-rank (Mantel-Cox) test].

We examined whether PROG treatment affected locomotor function of the TDP43^A315T^ mice by using the accelerating rotarod test. Of interest, we observed a significant difference in the rate of locomotor degeneration between the untreated and treated TDP43^A315T^ mice (Fig. 2C) over the entire treatment period. A quadratic linear regression was used to model the rate of loss of motor control over time (as measured by the change in mean latency period before fall from rotarod as mice age) with age (weeks), age^2^ (weeks×weeks), PROG treatment, and the interaction term (age^2^×PROG treatment) as covariates. PROG treatment significantly reduced the rate of loss of locomotor control (adjusted *P*=0.033 for interaction term) in PROG-treated compared with placebo-treated mice (Fig. 2C). An apparent 1.8-fold improvement in locomotor function of the PROG-treated TDP43^A315T^ mice was evident late in the study period, at 14.5 weeks (supplementary material Fig. S3B). However, this did not reach statistical significance when compared with age-matched placebo-treated TDP43^A315T^ mice.

## DISCUSSION

We have demonstrated that low levels of endogenous PROG were associated with FTD and that, within the subgroups, FTD-MND was particularly associated with low PROG (Table 1). PROG has previously been identified as a potential therapy for MND because high levels of the neurosteroid are associated with positive patient outcomes (Gargiulo Monachelli et al., 2011). Because individuals with MND also had higher overall levels of PROG, Gargiulo Monachelli et al. hypothesised that PROG was elevated in these individuals as a protective mechanism. A corollary of this hypothesis is that individuals who have low levels of PROG would be more likely to have disease, or have more severe clinical symptoms (Table 1). FTD-MND cases are characterised pathologically by nuclear clearing and cytoplasmic inclusions of TDP-43 (Mackenzie et al., 2010). Mutations in the *TARDBP* gene also result in mutant molecules that are depleted in the nucleus (Sreedharan et al., 2008), although it remains unclear whether mutant TDP-43 exerts its pathogenicity as a gain of novel function, or a loss of function via its depletion in the nucleus. We also showed that exogenous PROG can increase the level of nuclear TDP-43 in a neuroblastoma cell line (Fig. 1), supporting the concept that PROG might be able to reverse the pathogenic effects of depleted nuclear TDP-43.

PROG is an efficacious therapeutic agent in the Wobbler mouse model of MND. In these mice, PROG implants reduced both neuropathological lesions in the spinal cord and loss of motor functions, and increased survival rates (Gonzalez Deniselle et al., 2002). Moreover, the Wobbler mice have recently been shown to have elevated *TARDBP* gene expression and cytoplasmic inclusions of the TDP-43 protein (Dennis and Citron, 2009). We demonstrated a significant protective effect of PROG implants on locomotor control, but not survival, in TDP43^A315T^ mice (Fig. 2). There are a number of possible interpretations of the data. Firstly, most studies have shown that high levels of PROG, such as those achieved during a menstrual cycle or pregnancy (∼20,000–80,000 pg/ml), were required for its neuroprotective effects (Singh and Su, 2013). Therefore, although the levels of PROG achieved in our treated mice (5500 pg/ml, range 2700–9400 pg/ml) were comparable with other animal studies (Carroll et al., 2010) and the 9300 pg/ml used in our *in vitro* experiments (Fig. 1), the lack of protection on lifespan might be due to insufficiently high levels of PROG. Another possibility concerns the processing of exogenous PROG into its metabolites, such as 5α-pregnan-3α-ol-20-one, which have potential neuroprotective effects in a mouse model of Alzheimer’s disease (Frye and Walf, 2009). Because we did not monitor the levels of PROG or its bioactive metabolites, variability in the levels of these molecules during the treatment period could have masked some of the neuroprotective effects of the hormone. There is also the possibility that A315T mutant TBP-43 accumulates via an alternative mechanism, or represents a loss of protein function, to that for sporadic TDP-43 proteinopathies and that this mechanism is not amenable to PROG therapy. Finally, the TDP43^A315T^ mice might be dying due to reasons other than MND-like symptoms. A recent paper by Guo et al. showed that these mice developed significant gut problems, and consequently die before their MND phenotype is fully detectable (Guo et al., 2012). This is supported by our observation that the rotarod scores in these mice progressively decline but never dropped to zero (supplementary material Fig. S3), which is inconsistent with what is seen in other MND models (reviewed in Tsao et al., 2012). At present, there are currently no FDA-approved medications indicated for FTD treatment, and Riluzole, the only drug approved for the treatment of MND, yields a mere 3-month increase in survival when taken for 18 months (reviewed in Goodall and Morrison, 2006). Our clinical, cellular and animal data suggest that PROG might be an effective treatment for FTD-MND as well as MND.

Our data suggest that PROG might have therapeutic possibilities for clinical subgroups of FTD with Tau pathology, because low levels of the endogenous neurosteroid were also associated with increased risk of CBS (Table 1). Similar to the reported positive correlation between PROG levels and MND symptoms (Gargiulo Monachelli et al., 2011), we also observed that higher levels of endogenous PROG (>195 pg/ml) were correlated with lower disease severity in individuals with CBS. Although we did not observe similar effects in the PSPS or PNFA clinical subgroups, these subgroups are associated with neuronal Tau pathology, whereas in CBS Tau accumulation occurs primarily in glial cells (Kouri et al., 2011). We further observed that exogenous PROG significantly downregulated levels of total Tau protein in a cell culture model (Fig. 1), and might be the mechanism by which PROG affects the disease parameters of certain FTD subgroups. This was supported by the observation that PROG level was a significant negative predictor of serum Tau level in individuals with available data (supplementary material Fig. S1). However, this result requires confirmation from a study with larger sample size and longitudinal measurements of Tau and PROG levels. Longitudinal studies effectively adjust for inter-individual variability in hormone levels and have identified consistent changes in the levels of blood-based biomarkers with disease progression (reviewed in Henry et al., 2013). Although no studies have demonstrated an effect of PROG on Tau expression in glial cells, elevated PROG during pregnancy is thought to mediate remission of symptoms in women with multiple sclerosis via its effect on glial cells, which express the progesterone receptor (Kipp et al., 2012). The efficacy of PROG has been demonstrated in a triple-transgenic mouse model for neurodegeneration that carries a mutant Pro301Leu *MAPT* gene (Carroll et al., 2010). PROG treatment in these mice reduced the levels of age-related hyperphosphorylated Tau. In conclusion, our data suggest that low PROG levels occur in certain subgroups of FTD (TDP-43 proteinopathies, and those with significant glial Tau accumulation), and that the level of PROG is related to the severity of disease in those with significant glial Tau accumulation. Our cellular and animal model data further support the hypothesis that PROG acts directly by modulating the levels of the two key pathological molecules, TDP-43 and Tau, and thus could be a viable therapeutic target for subgroups of FTD, as has been proposed for MND.

## MATERIALS AND METHODS

### Chemicals

PROG (4-pregnene-3,20-dione) was obtained from Sigma-Aldrich (MO). PROG pellets for implants (200 mg pellet designed to be released over a 90-day period) were obtained from Innovative Research of America (FL).

### Clinical classification

Patients were selected from the FRONTIER database (http://www.neura.edu.au/frontier). All patients underwent a full neurological and cognitive assessment by an experienced neurologist (J.R.H.). All patients were classified according to the clinical variants of FTD at presentation, or within 6 months of presentation (Gorno-Tempini et al., 2011; Rascovsky et al., 2011). Patients were included in this study based on their current clinical diagnosis, which, from previous clinicopathological studies (Hodges et al., 2004; Schofield et al., 2012), have been demonstrated to be highly predictive (>75% accuracy) for either Tau or TDP-43 neuropathological inclusions at autopsy. We employed the FRS, which is capable of staging disease severity in FTD based upon functional dependence and behavioural changes (Mioshi et al., 2010). Five severity stages (very mild to very severe) were identified in this cohort and operationalised based upon a 30-item questionnaire that gathers information on the patient’s behaviour, ability to perform everyday tasks (such as shopping, preparing food and eating) and mobility. Written informed consent was obtained for all participants in this study and the study was approved by the Human Research Ethics Committees of the South Eastern Sydney and Illawarra Health Service-Northern Hospital Network, and the University of New South Wales.

### Serum PROG collection and analysis

Whole blood was collected, left at room temperature for 30 minutes to clot, then centrifuged at 395 ***g*** for 10 minutes at 4°C. The serum layer was removed and stored frozen at −80°C. Control serum samples from 91 individuals with no neurological disorder were selected from a serum bank at Neuroscience Research Australia stored at −80°C. PROG levels were determined by South Eastern Area Laboratory Services (SEALS) at the Prince of Wales Hospital, Sydney, Australia. The PROG assay was performed with the IMMULITE 2000 XPi analyser and Immulite Progesterone reagent kit (Siemens, Erlangen, Germany). The mean inter-assay variability was 9.2% and mean intra-assay variability was 11.8%. A PROG level of 195 pg/ml was the lowest limit of sensitivity for the assay.

### Human total Tau ELISA

Tau (Total) Human ELISA kit (Life Technologies, VIC, Australia) was used to determine serum Tau levels according to the manufacturer’s instructions. Standard curve using recombinant Tau was used to adjust for inter-assay variability. Intra-assay variability was <1%.

### Determination of total Tau and TDP-43 levels and subcellular localisation by western blotting

PROG was dissolved in 100% ethanol and diluted to final concentration in cell culture medium prior to use. SK-N-MC (ATCC HTB 10) cells were exposed to different doses of PROG for 48 hours prior to western blot analyses of Tau and TDP-43 protein levels as described previously (Luty et al., 2010). Rabbit polyclonal antibodies were used to detect the TDP-43 protein (rabbit polyclonal ab41881, Abcam, MA; diluted 1:2000) and Tau protein (mouse monoclonal clone 5E2, Millipore, CA; diluted 1:2000). Differences in protein levels between different samples were normalised using β-actin levels (mouse monoclonal clone C4, Abcam, Millipore, CA; diluted 1:10,000). Protein bands were visualised using the Chemiluminescent HRP substrate (Millipore, MA) and were quantified using the Chemidoc system (Bio-Rad, CA).

### Behavioural phenotyping

All animal procedures were approved by the University of New South Wales (Australia) and University of Melbourne (Australia) Animal Ethics committees. Hemizygous TDP43^A315T^ (Prnp-TARDBP*A315T) mice were obtained from Jackson Laboratory (ME) and a colony maintained by breeding with C57BL/6 mice. Animals were housed in cages containing 2–5 mice. The cages were lined with standard bedding material and kept under conditions of controlled humidity and temperature (22°C), with lights on from 07:00 to 19:00 hours. The mice had *ad libitum* access to food and water. At 5.5 weeks of age, TDP43^A315T^ mice and non-transgenic littermates were implanted with a 200 mg pellet of PROG or placebo control pellet under the skin under light isoflurane anesthesia. For this study, 21 non-transgenic male mice (10 placebo-treated and 11 with PROG pellet implants) and 27 TDP43^A315T^ male mice (14 placebo-treated and 13 treated) were used. Non-transgenic littermates of the TDP43^A315T^ mice were used as the non-transgenic controls, and the placebo and PROG treatments were allocated randomly across litters. Efficacy of the PROG treatment was checked by measuring PROG levels in fresh brain tissue using a Progesterone Enzyme Immunoassay kit (Cayman Chemical, MI). Therapeutic efficacy of the PROG treatment was examined by assessing locomotor function of the mice twice weekly using the accelerating rotarod test. Mice were placed on the rotating rod, which accelerated linearly from 4 to 40 rpm over 5 minutes, and the time each mouse remained on the rod (latency to fall) recorded.

### Western blot analysis of ChAT protein

Spinal cords from TDP43^A315T^ mice (*n*=9) at early disease stage (12 weeks) and age-matched wild-type mice (*n*=9) were dissected and mechanically homogenized in phosphate buffered saline containing 1:50 phosphatase inhibitor cocktail, 1:100 protease inhibitor and 1:20 DNase I (Roche Applied Sciences, NSW, Australia), followed by centrifugation at 18,000 ***g*** for 3 minutes at 4°C. Soluble proteins were collected from the supernatant fractions. ChAT protein levels were determined by western blot analysis (goat polyclonal AB144P, Millipore, MA; diluted 1:500). Differences in protein levels between different samples were normalised using GAPDH levels (rabbit polyclonal D16H11, Cell Signaling, MA; diluted 1:5000). Intensities of chemiluminescence bands were quantified using LAS-3000 Image reader (Fujifilm, CT). Group differences in normalised ChAT levels from western blot analyses were examined using the non-parametric Wilcoxon test.

### Statistics

Endogenous PROG levels were coded as a categorical variable, with low-expressors (≤195 pg/ml) assigned a value of 0 and individuals with detectable levels of PROG assigned a value of 1. This was to avoid assumptions regarding the absolute value of PROG in individuals with undetectable PROG levels (≤195 pg/ml). Binary logistic regression was used to examine the relationship between PROG levels and disease status (binary outcome). Multiple linear regression was used to examine the correlation between PROG levels and the FRS (continuous variable). *P*-values obtained from the examination of the relationship between PROG and FTD clinical subgroups were corrected for multiple testing using the False Discovery Rate method (Benjamini and Hochberg, 1995). Differences in normalised Tau and TDP-43 levels from western blot analyses were examined using the non-parametric Wilcoxon test. Quadratic linear equation was used to model accurately the steep decline in loss of locomotor control with age when mice reached end-stage of disease, because the rate of decline was not linear. Model fitting regression analysis demonstrated that the improvement in correlation (change in *r*^2^=0.695) between rotarod scores and age (weeks) was significant (*P*=5.5×10^−9^) when a quadratic term for age^2^ (weeks×weeks) was included in the equation. Differences in absolute rotarod scores at specific ages were examined using a 1-way ANOVA with Dunn’s multiple comparisons post-test. Differences in survival curves were examined using the log-rank (Mantel-Cox) test. All statistical analyses were performed in SPSS v. 20 (IBM, Armonk, NY).

## Supplementary Material

Supplementary Material
