# Supplementary material for: Endogenous progesterone levels and frontotemporal dementia: modulation of TDP-43 and Tau levels in vitro and treatment of the A315T TARDBP mouse model
Source: Dis Model Mech. 2013 Jun 20;6(5):1198–204. doi: 10.1242/dmm.011460 (PMC3759339; doi:10.1242/dmm.011460)
Supplement: Supplementary Material [file supp_6_5_1198__index.html]

Endogenous progesterone levels and frontotemporal dementia: modulation of TDP-43 and Tau levels in vitro and treatment of the A315T TARDBP mouse model — Endogenous progesterone levels and frontotemporal dementia: modulation of TDP-43 and Tau levels in vitro and treatment of the A315T TARDBP mouse model — Supplementary Material 

# Endogenous progesterone levels and frontotemporal dementia: modulation of TDP-43 and Tau levels *in vitro* and treatment of the A315T *TARDBP* mouse model

## 

**Files in this Data Supplement:**

- **Supplementary Material PDF**
