# Supplementary material for: Endogenous progesterone levels and frontotemporal dementia: modulation of TDP-43 and Tau levels in vitro and treatment of the A315T TARDBP mouse model
Source: Dis Model Mech. 2013 Jun 20;6(5):1198–204. doi: 10.1242/dmm.011460 (PMC3759339; doi:10.1242/dmm.011460)
Supplement: Supplementary Material [file supp_011460_DMM011460.pdf]

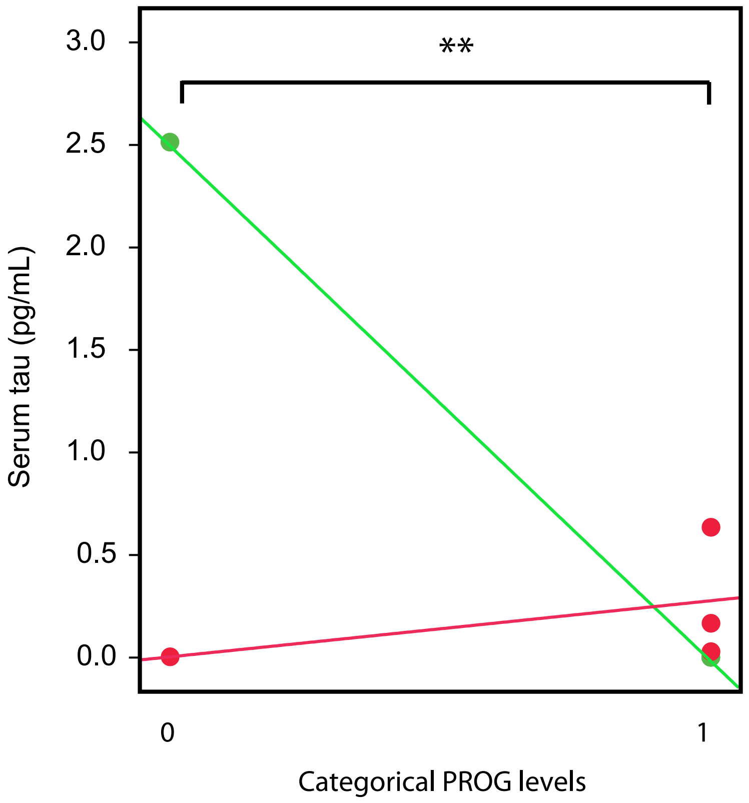

Supplementary Figure S1: Scatter plot of serum progesterone (PROG) levels and serum tau levels. Individuals with low PROG levels (< 195 pg/mL) were coded with a value of 0, and everyone else were coded as 1. Disease status is coded as a dummy variable with '0' for controls (green) and '1' for FTD patients (pink). Multiple regression analysis shows that PROG levels were significant predictors for tau levels when an interaction term (disease x PROG levels) was included in the regression equation. Significance level is indicated (** $p < 0.001$).

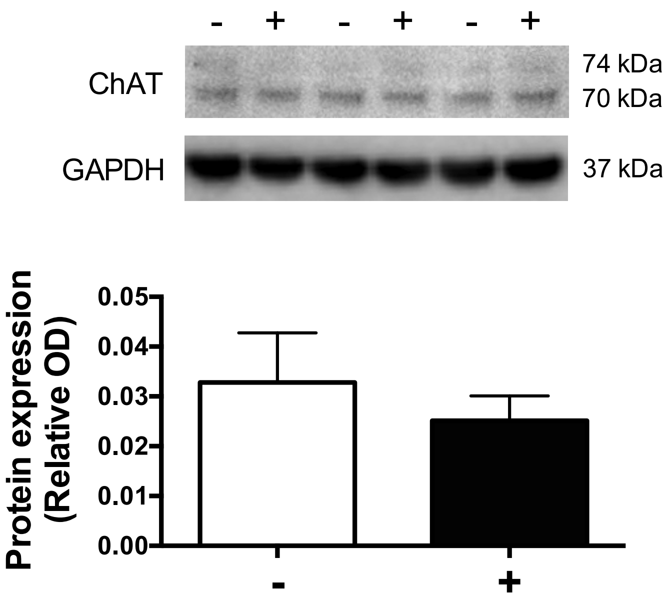

Supplementary Figure S2: Levels of spinal cord choline acetyltransferase (ChAT) of untreated wildtype (-) and TDP43^{A315T} mutant mice (+). Western blot analysis revealed the two main 74 kDa and 70KDa isoforms of ChAT in spinal cord tissue (upper panel). Quantification of normalized ChAT in wildtype and transgenic animals show no significant differences in ChAT levels (Wilcoxon statistic = 80, $p > 0.05$) (lower panel).

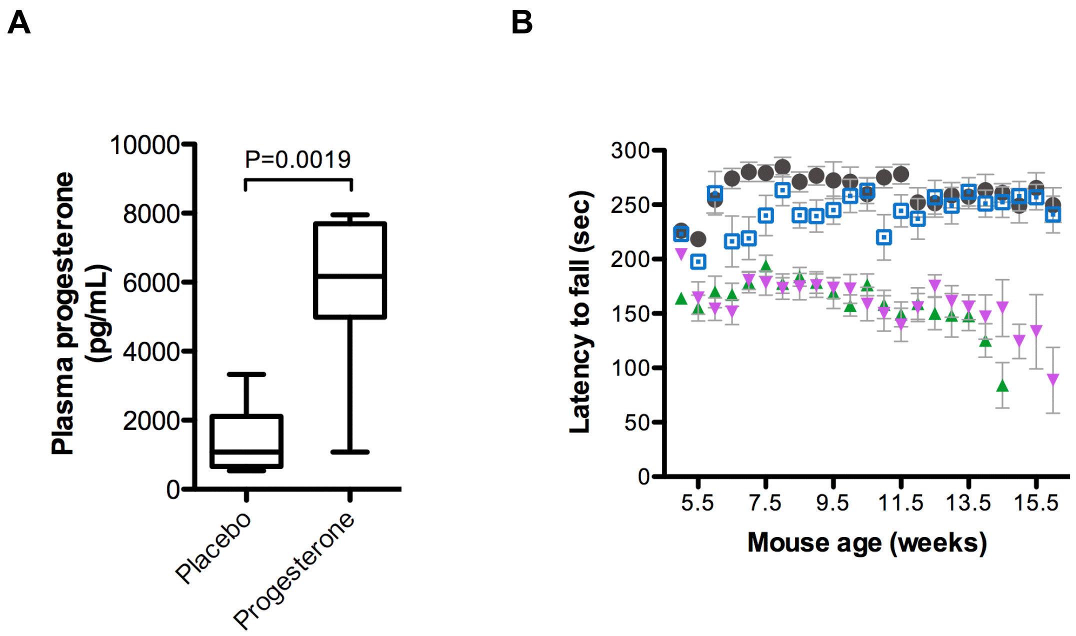

Supplementary Figure S3: Effect of progesterone (PROG) treatment on locomotor control of TDP43^{A315T} mutant mice. A) Levels of serum PROG of treated and untreated wildtype mice. Note that mean level of 5870 pg/ml PROG is equivalent to 19 nM PROG. B) Mean values for absolute time spent on rotarod were not significantly increased by PROG treatment of TDP43^{A315T} mutant mice (pink triangle) or wildtype (blue squares) compared with age-matched placebo treated mutant (green triangles) or wildtype (black circles). Note that the transgenic mice (PROG and placebo treated) had significant early locomotor deficits compared with non-transgenic (PROG and placebo treated) animals even at 6 weeks (1-way ANOVA with Dunn's multiple comparisons post-test, $p < 0.05$).
